# 10-Year Longitudinal Changes in Fitness Parameters in Physiotherapy Students

**DOI:** 10.1155/2020/7154797

**Published:** 2020-07-07

**Authors:** Andrzej Lewandowski, Jadwiga Sarwińska, Marcin Siedlaczek, Zuzanna Piekorz

**Affiliations:** Chair of Physiotherapy, Faculty of Health Sciences, Ludwik Rydygier Collegium Medicum in Bydgoszcz, Nicolaus Copernicus University, Poland

## Abstract

**Introduction:**

The aim of this study is to examine changes in the chosen morphological characteristics, motor conditioning, and coordination skills in physiotherapy undergraduates at a medical university in Bydgoszcz in the first decade of the millennium. We assume prevalence of a secular trend in values of morphological characteristics; however, characteristics of motor skills, particularly fitness levels, will remain relatively unchanged.

**Materials and Methods:**

The study included young people studying physiotherapy at the medical university of Bydgoszcz 2001-2010. Basic morphological features, including waist and hip circumferences, were measured, and BMI and WHR were calculated. The Cooper test and marching over a distance of two kilometers were employed to determine endurance; right and left spinning coordination was assessed by Starosta test. Calculated Mollison indicators were used in the evaluation of dimorphic differences in subsequent observation time. *Statistics*. Data are presented as mean with the standard deviation. Groupwise comparison was performed with Student's *t*-test and linear relationships with Pearson's *r*. Linear covariance models (ANCOVA) were built as theoretical models. Statistical significance was set at alfa = 0.05. Based on the correlation between the data and the corresponding normal score used, the Shapiro-Wilk test is the best choice for testing the normality of data. Variables were excluded if they exceeded the tolerance level for multicollinearity. Analysis was performed with Statistica 10.

**Results:**

The study demonstrated a rising number of male students (from 13,3% to 39,2%, chi^2^ = 10, 13; *p* = 0,001) and a decrease in age of students overall but no significant changes (from 22, 25 ± 0, 75 to 20, 42 ± 1, 7; *F* = 32, 9; *p* < 0,001) in their morphological characteristics and their dimorphic differences (average results for males: BMI, from 22, 25 ± 1, 94 to 26, 27 ± 3, 56; WHR index, from 0, 78 ± 0, 06 to 0, 85 ± 0, 06; average results for females: BMI, from 20, 79 ± 2, 11 to 22, 19 ± 3, 83; WHR index, from 0, 7 ± 0, 04 to 0, 75 ± 0, 15). An overall improvement in endurance was found; however, coordination, especially amongst women, had deteriorated (turn right, from 377,1 ± 48, 1 to 343,1 ± 23, 3; turn left, from 375,0 ± 5, 61 to 345,6 ± 43, 1). We observed multidirectional changes in the analyzed motor skills and most of them were statistically significant.

**Conclusions:**

Longitudinal study of physiotherapy students revealed no differences in morphological and dimorphic characteristics and multimodal changes in assessed motor skills, thus highlighting a need for further research into the identification of their causes. Moreover, a requirement for entry motor skills assessment and curriculum reorientation towards delivery of a broader scope of physical education was suggested.

## 1. Introduction

Physiotherapy was recognized as an integral part of medicine during the postwar period. The continuous dynamic development of this field, particularly with regard to methods used and areas leading to professional fulfillment, is owed to its effectiveness. Physiotherapy degree courses were initially delivered by physical education colleges. Years later, following on an example set by the medical university in Bydgoszcz at the end of the 1990s, other medical colleges introduced the course as well. Nowadays, an increased popularity and appeal of physiotherapy as a career choice caused that degree courses are offered by many public and nonpublic universities, even those not specialized in health sciences. The above arguments and market mechanisms in the education field mean that despite expectations of high physical fitness level of the physiotherapist, most of universities do not use motor selection for studies [1, 2]. Thus, an increased number in undergraduates who do not pay attention to the level of their own physical fitness adequate to the profession is observed.

A gap in research on fitness levels in physiotherapy students does not allow for an adequate estimation of its level. Limited data and empiric experience indicate adverse effects on changes in fitness composition and level of performance in the work role, caused by lifestyle changes and secular trends in anthropometric parameters [3–5]. The available literature highlights that physiotherapy students have a similar or higher level of physical fitness compared to other students [6–8]. Our study demonstrates more advantageous fitness parameters in physiotherapy undergraduates [9–12] even though the majority of them take the course at universities other than physical education universities.

Physiotherapists are expected to employ high levels of physical activity; a similar degree of fitness and motor experiences allows for implementation of professional standards [13]. This is the most probable reason for outlining endurance and motor coordination as key determinants in successful delivery of the work role [1, 2]. Their level is determined by many factors, both endogenous and exogenous as well as body structure [14–18]. Civilizational development shapes the socioenvironmental conditions in population and thus impacts their subsequent biological effects [4]. Consequently, it causes changes in patients' healthcare and education system for prospective employees. Thus, a long-term change in the biological value in physiotherapy students, measured by morphological characteristics and fitness and motor skill, can be assumed.

Considering the above and a growing number of physiotherapy students, it was imperative to assess their physical aptitude. Thus, the aim of this study is to examine changes in the chosen morphological characteristics, motor coordination, and conditioning skills in physiotherapy undergraduates at the medical university in Bydgoszcz in the first decade of the millennium. We assumed prevalence of a secular trend in the values of morphological characteristics and no major differences in motor skills, particularly conditioning. With extensive longitudinal study, an attempt to identify temporal changes in these parameters was made, which may have provided arguments for the introduction of changes in the curriculum and in entry requirements for physiotherapy degree courses.

## 2. Material and Method

The research included young people studying physiotherapy at the medical university of Bydgoszcz in 2001-2010. Data was collected during Exercise Science classes, initially taught in the third year and later delivered in the second year of a comprehensive five-year master courses and first year of full-time bachelor degree studies. In 2001-2007, third year students following the comprehensive master degree pathway were included; they had been required to pass a fitness test upon entry and in three consecutive years. In 2007-2009, second year bachelor degree students following a similar examination framework were analyzed. In 2009 and 2010, the subjects were first year undergraduates not requiring a fitness test for admission.

The research outcomes of the third-year master degree and second-year bachelor degree students examined in 2007 were considered collectively, similar to the results in first and second year undergraduates in 2009. During the research period, the curriculum underwent numerous modifications; however, the exercise and movement classes and teaching hours remained unchanged.

Thus, at the end of each academic year, body height and mass were measured as basic morphological features in addition to waist and hip circumferences. The BMI and WHR indexes were calculated separately for each participant [19]. Endurance, as a part of gross motor skills, was measured by the Cooper test and a march over a distance of two kilometers; its levels are strongly correlated with VO2max. Starosta's test was employed for the assessment of right and left spinning coordination [20–22].

Calculated Mollison indicators, commonly used in in different fields of physical anthropological studies, were used in the evaluation of dimorphic differences in assessed characteristics in consecutive observation years [23, 24]. All measurements were carried out by a specialist research team in the same conditions and in accordance with testing procedures. Anthropometric assessment and conditioning tests were performed once; the best two scores of the latter were recorded. Overall the results of 217 male and 538 female students were obtained which covered 95% of the researched population.

### 2.1. Statistics

Data are presented as mean with the standard deviation. Groupwise comparison was performed with Student's *t*-test and linear relationships with Pearson's *r*. Linear covariance models (ANCOVA) were built as theoretical models. Statistical significance was set at alfa = 0.05. Based on the correlation between the data and the corresponding normal score used, the Shapiro-Wilk test is the best choice for testing the normality of data. Variables were excluded if they exceeded the tolerance level for multicollinearity. Analysis was performed with Statistica 10.

## 3. Results

**Figure 1:**
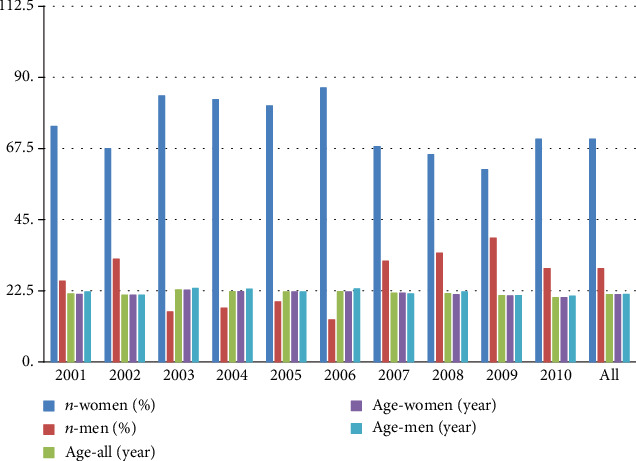
Demographic characteristics of participants.

Demographic characteristics of participants are shown in Table 1 and Figure 1.

The research proved a substantial reduction in the female participation (chi^2^ = 10, 13; *p* = 0,001) and a significant decrease in participants' age at the time of research (*F* = 32, 9; *p* < 0,001). No meaningful correlation between age and gender in participants was noted (*F* = 0, 70; *p* = 0,406). Anthropometric characteristics of participants are shown in Tables 2 and 3. In both groups, the sizes of somatic features measured in individual years of observation were similar and their changes were multidirectional.

Occurrence of significant changes in anthropometric characteristics in subsequent years was assessed. Due to aforementioned changes in the gender structure and statistical characteristics in participants' age, these two parameters were also included in the ACNOVA covariance analysis.

The characteristics of the obtained covariance models are presented in Table 4.

Research demonstrates no significant effect of the test year on any of the analyzed anthropometric parameters. Older students are characterised by significantly higher values of body mass, BMI index, and waist circumference and substantially lower body height. All anthropometric parameters in women assumed significantly lower values.

The results of the fitness tests in the study participants are presented in Table 5. For both genders, the results of motor skills tests were different. Their changes in individual years of observation were clear and as in somatic features multidirectional.

Occurrence of significant changes in fitness tests in consecutive years was also assessed. Due to aforementioned changes in the gender structure and statistical characteristics in participants' age, these two parameters were also included in the ACNOVA covariance analysis. The anthropometric characteristics were not considered since a significant correlation between gender and age had been demonstrated.

The characteristics of the obtained covariance models are presented in Table 6.

Covariance analysis revealed significantly higher scores in the Cooper test for consecutive years. In addition, we noted two independent factors significantly increasing a probability of a lower test outcome: older age and of female gender, which proved to be a sole factor substantially prolonging walking time at 2 km.

Substantial change in right spinning coordination in participants assessed in consecutive years was also found. Another independent factor contributing to poor results in this test was female gender; it also decided lower outcomes in left spinning coordination.

**Figure 2:**
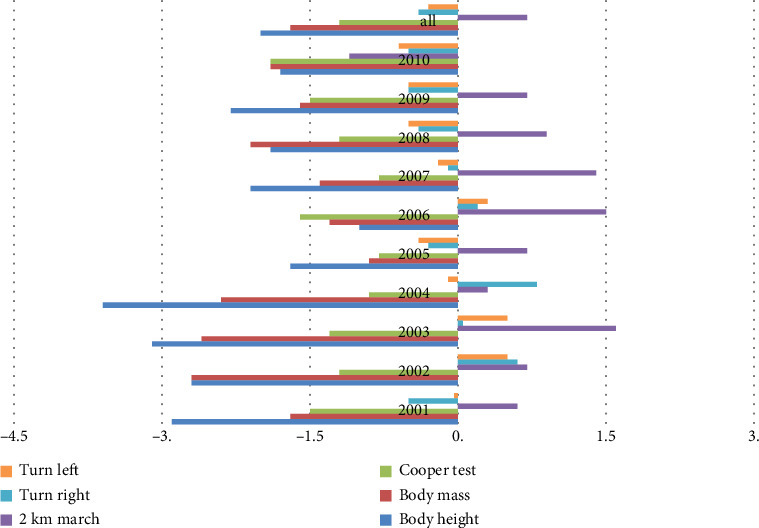
Graphic characteristics of the calculated Mollison index.

The value of the Mollison index of the examined characteristics in male and female students, allowing for the assessment of differences in consecutive research years, is presented in Table 7 and Figure 2.

Small gains were found in dimorphic differences in body height and fitness levels, particularly in coordination.

## 4. Discussion

The presented study allows for basic observations and conclusions to be drawn, which however, requires a broader discussion and further reference to available literature. The outcomes of our longitudinal research indicate changes in gender composition and participants' age as well as in assessed motor characteristics.

Predominance of female adolescents which decreased over the observation period highlights feminization of medical professions in Central and Eastern Europe [25]. Moreover, many of the early research stage participants, mostly women, had previously been unsuccessful in applying for medical studies. Later changes in the recruitment process as well as a greater number of degree courses offered by institutions could have impacted on the gender structure and age in the examined students. Most probably, observed changes in both characteristics are consequential to various social factors such as opportunity to enter an interesting, well-paid profession with numerous options to work abroad brought about by an aging population and increasingly sedentary lifestyle, as well as work in areas of employment that require more physical effort.

It is also possible that the reason for changes in students' age factor was blending of full-time and part-time programmes that allowed previously unsuccessful applicants to take a degree in physiotherapy. In either instances, abandonment or nonperformance of a fitness test as entry requirement could have been an important factor.

These assumptions are however difficult to confirm with reliable scientific research results due to lack of broader publications on physiotherapy undergraduates.

No significant changes were observed in anthropometric characteristics in consecutive years of observation, although previous longitudinal studies and predictions for subsequent ones indicate maintenance of secular changes [26, 27]. Thus, the lack of significant long-term changes in morphological features in young physiotherapy students may indicate diminishment of the secular trend phenomenon in this group of academic youth, and consequently self-driven attitude towards the field of study and chosen profession. It should be noted, however, that older participants from the same year are characterised by smaller average body height and greater body mass. The phenomenon of larger waists in older students suggests adverse lifestyle-related changes occurring with age rather than as a result of a secular trend. This supposition indirectly confirms similar morphological and motor characteristics in adolescents of different ages studying medical sciences and an evidence of secular trend in basic anthropometric features in Polish conscripts where strict and directional selection are not performed [26, 28].

Multimodal changes in motor characteristics were also observed. The improvement in results of the Cooper test is a positive phenomenon, but lack of progress in the march test demonstrates the influence of factors other than efficiency potential on endurance in physiotherapy students. The key factors could be personal and environmental aspects such as an increased popularity of running sports, or to a lesser degree walking, which encourage persons' efficiency potential and personal characteristics determining endurance.

It is possible, however, that observed improvement in students' performance resulted from their conscious effort to maintain good health, as well as attempts to satisfy training and job requirements.

Thus, it can be stated that in this respect male physiotherapy students are better prepared to implement professional standards requiring considerable physical effort as had been confirmed by analysis of dimorphic differences in examined characteristics. The probability of getting lower scores in both endurance tests by older students can be explained by an adverse impact of higher body mass, and thus higher BMI. Relative lower potential in women endurance caused by physiological processes may also affect this result [29].

Negative change in coordination appears somewhat worrying as it manifests itself in the nervous system and impacts on the quality of performing motor tasks [30].

Deterioration of results over time may therefore signify a regression in quality of movements and motor skills in prospective therapists, possibly as a result of participation in a limited variety of activities, often narrowed down to running-based sports. Such an assumption indirectly justifies a significant deterioration in the scope of right spinning, performed at a lower frequency in both, everyday life and many sports competitions.

However, it needs to be noted that the tests used in our assessment measures specific, power-based coordination skills and its accuracy is very high in the case of figure skaters, gymnasts, and dancers [22]. Thus, observed adverse changes in coordination need to be confirmed by additional assessments using less specific tests.

Detection of multimodal changes in motor characteristics, included in various groups of motor skills, can therefore indicate changes in the fitness structure in future physiotherapists [13]. Therefore, deterioration of coordination level while sustaining values of basic morphological features, also observed in long-term studies of physical education students in Cracow, seems worrying, especially in women whose participation in studies and occupation clearly prevails [31]. This conclusion is also confirmed by research outcomes demonstrating importance of a high level of both motor properties in the successful fulfillment of the work role, and the fact that a wide range of motor skills, manifested by motor coordination level, are fundamental to physiotherapist in the field of kinesiotherapy [4].

When discussing the study outcomes, we shall mention the potential limitations that could affect the final result. One of them may be combining student groups from different years and study pathways that happened to take the module in the very same academic year. Implementation of such proceedings was, however, justified by the results of our previous research revealing that students of different years pursuing education at a medical school are already shaped with regard to morphological traits and motor skills [19] and that the merged student groups were required to pass a fitness exam as a part of recruitment procedure.

The analysis of results relating to covariance models also confirms that changes in motor characteristics were influenced by factors other than the measured anthropometric features. Thus, the basis for long-term changes in the biological value in physiotherapy students, determined by examined morphological and motor characteristics, should be seen in other socioenvironmental conditions, such as differences in physical fitness levels in physiotherapy students at a medical university and physical education college, as well as undergraduates from other medical and health sciences faculties [7, 9–12]. The above assumption justifies the need to extend the current analysis with changes in greater numbers of fitness parameters and to continue observation using the conclusions below.

## 5. Conclusions

Long-term studies showed an increase in the number of male physiotherapy students, decrease in age of students overall, and lack of significant changes in morphological characteristics which indicates diminishing of a secular trend in academic students' height and body mass. The study also found gains in the outcomes of the Cooper test and decrease in results of rotation coordination, particularly in women, that prove changes in the structure of physical fitness. It may have been caused by being aware of future job requirements or stemmed from trends for fitness, particularly running. Presented observations and temporal changes in dimorphic differences in the examined characteristics demonstrate that male physiotherapy students are better prepared to satisfy professional standards that require considerable physical effort. This justifies requirement for an entry assessment of motor skills and curriculum reorientation towards delivering a broader scope of physical education.

## Figures and Tables

**Table 1:** Demographic characteristics in/of participants.

Years of study	*n* (%)			Age (mean ± SD)		
	All	Women	Men	All	Women	Men
2001	47	35 (74,5)	12 (25,5)	21, 55 ± 1, 32	21, 37 ± 0, 73	22, 08 ± 2, 27
2002	40	27 (67,5)	13 (32,5)	21, 13 ± 0, 56	21, 11 ± 0, 51	21, 15 ± 0, 69
2003	38	32 (84,2)	6 (15,8)	22, 79 ± 1, 28	22, 69 ± 1, 26	23, 33 ± 1, 37
2004	47	39 (83,0)	8 (17,0)	22, 32 ± 1, 11	22, 18 ± 0, 72	23, 00 ± 2, 14
2005	58	47 (81,0)	11 (19,0)	22,10 ± 0,48	22,11 ± 0,52	22, 09 ± 0, 30
2006	60	52 (86,7)	8 (13,3)	22, 25 ± 0, 75	22, 12 ± 0, 43	23, 13 ± 1, 55
2007	116	79 (68,1)	37 (31,9)	21, 65 ± 0, 91	21, 70 ± 0, 85	21, 54 ± 1, 02
2008	61	40 (65,6)	21 (34,4)	21, 57 ± 1, 78	21, 30 ± 0, 72	22, 10 ± 2, 84
2009	176	107 (60,8)	69 (39,2)	20, 98 ± 0, 89	20, 95 ± 0, 87	21, 01 ± 0, 93
2010	112	79 (70,5)	33 (29,5)	20, 42 ± 1, 70	20, 27 ± 1, 20	20, 79 ± 2, 51
All	755	589 (70,5)	246 (29,5)	21, 35 ± 1, 33	21, 32 ± 1, 11	21, 41 ± 1, 75

*n* (%): numerical and percentage characteristics of the size of student groups surveyed; mean: average age of student groups surveyed; SD: standard deviation.

**Table 2:** Statistical characteristics (mean ± SD) of body mass, height, and BMI in participants.

Years of study	Body mass (kg)		Body height (cm)		BMI (kg/m^2^)	
	Women	Men	Women	Men	Women	Men
2001	59, 11 ± 7, 49	77, 33 ± 10, 57	165,74 ± 5, 15	180,70 ± 5, 20	21, 51 ± 2, 52	23, 61 ± 2, 33
2002	60, 78 ± 7, 37	75, 62 ± 5, 39	169,25 ± 6, 65	183,51 ± 5, 54	21, 23 ± 2, 50	22, 5 ± 1, 86
2003	59, 81 ± 9, 78	87, 17 ± 10, 61	165,89 ± 7, 37	182,33 ± 5, 30	21, 69 ± 2, 99	26, 27 ± 3, 56
2004	58, 56 ± 9, 01	80, 88 ± 9, 42	166,07 ± 6, 34	180,09 ± 3, 89	21, 21 ± 2, 79	25, 04 ± 3, 67
2005	59, 91 ± 11, 44	68, 64 ± 9, 83	164,15 ± 5, 90	175,25 ± 6, 40	22, 19 ± 3, 83	22, 25 ± 1, 94
2006	59, 60 ± 6, 79	77, 25 ± 13, 88	165,44 ± 5, 07	177,38 ± 11, 84	21, 77 ± 2, 24	24, 45 ± 2, 52
2007	61, 59 ± 9, 47	75, 44 ± 10, 05	167,82 ± 6, 32	179, 60 ± 5, 69	21, 84 ± 2, 91	23, 39 ± 2, 91
2008	58, 55 ± 6, 73	79, 00 ± 9, 81	166,69 ± 5, 94	180,31 ± 7, 18	21, 06 ± 1, 95	24, 34 ± 2, 96
2009	60, 36 ± 8, 95	77, 10 ± 10, 62	166,72 ± 6, 23	180,99 ± 6, 08	21, 68 ± 2, 68	23, 49 ± 2, 55
2010	58, 49 ± 7, 46	76, 33 ± 9, 52	167,61 ± 6, 10	179,75 ± 6, 82	20, 79 ± 2, 11	23, 57 ± 1, 93
All	59, 85 ± 8, 76	76, 79 ± 10, 07	166,63 ± 6, 08	179,95 ± 6, 54	21, 53 ± 2, 78	23, 69 ± 2, 62

Body mass: arithmetic mean and standard deviation of body mass of examined student groups; body height: arithmetic mean and standard deviation of body height of examined student groups; BMI: arithmetic mean and standard deviation of the body mass index of the examined student groups.

**Table 3:** Statistical characteristics (mean ± SD) of WHR index and waist and hip circumference.

Years of study	Waist circumference (cm)		Hip circumference (cm)		WHR index	
	Women	Men	Women	Men	Women	Men
2001	69, 83 ± 8, 66	79, 75 ± 6, 71	94, 14 ± 8, 61	99, 58 ± 6, 47	0, 75 ± 0, 15	0, 80 ± 0, 03
2002	67, 04 ± 7, 71	75, 73 ± 4, 46	93, 81 ± 6, 63	92, 31 ± 5, 45	0, 71 ± 0, 07	0, 82 ± 0, 06
2003	66, 24 ± 6, 32	85, 75 ± 8, 82	94, 50 ± 7, 21	100,25 ± 6, 16	0, 70 ± 0, 05	0, 85 ± 0, 06
2004	67, 26 ± 8, 29	83, 75 ± 10, 90	93, 34 ± 6, 39	97, 69 ± 5, 62	0, 72 ± 0, 07	0, 86 ± 0, 07
2005	66, 30 ± 7, 48	72, 73 ± 6, 42	94, 13 ± 8, 38	93, 00 ± 4, 58	0, 70 ± 0, 04	0, 78 ± 0, 06
2006	67, 35 ± 5, 57	79, 88 ± 5, 96	92, 95 ± 5, 03	95, 50 ± 5, 42	0, 72 ± 0, 04	0, 84 ± 0, 04
2007	65, 70 ± 5, 88	77, 35 ± 8, 15	92, 99 ± 5, 71	93, 86 ± 6, 01	0, 71 ± 0, 04	0, 82 ± 0, 04
2008	65, 93 ± 5, 89	81, 05 ± 8, 44	91, 93 ± 5, 36	97, 00 ± 5, 59	0, 72 ± 0, 04	0, 83 ± 0, 06
2009	67, 02 ± 6, 53	77, 38 ± 7, 10	92, 17 ± 5, 71	94, 84 ± 5, 32	0, 73 ± 0, 05	0, 82 ± 0, 05
2010	66, 15 ± 6, 02	77, 58 ± 7, 53	92, 20 ± 5, 77	94, 85 ± 5, 40	0, 72 ± 0, 04	0, 82 ± 0, 05
All	66, 84 ± 6, 88	78, 19 ± 7, 56	93, 01 ± 6, 39	95, 25 ± 5, 68	0, 72 ± 0, 06	0, 82 ± 0, 05

Waist circumference: arithmetic mean and standard deviation of waist circumferences of the examined student groups; hip circumference: arithmetic mean and standard deviation of hip circumferences of examined student groups; WHR index: arithmetic mean and standard deviation of the waist-hip ratio of examined student groups.

**Table 4:** Characteristics of covariance models assessing the impact of research year, gender, and age of participants on their anthropometric characteristics.

Feature	*β*	SD	95% CI	*p*
Body mass (*R*^2^ = 0,420; *p* < 0,001)				
Age	0,071	0,029	([0,014]-[0,127])	0,014
Year	0,026	0,029	([-0,031]-[0,082])	0,378
Female	-0,640	0,027	([-0,692]-[-0,588])	<0,001
Body height (*R*^2^ = 0,493; *p* < 0,001)				
Age	-0,070	0,027	([-0,123]-[-0,017])	0,009
Year	-0,017	0,027	([-0,070]-[0,036])	0,523
Female	-0,703	0,025	([-0,752]-[-0,654])	<0,001
BMI (*R*^2^ = 0,134; *p* < 0,001)				
Age	0,148	0,035	([0,079]-[0,217])	<0,001
Year	0,048	0,035	([-0,022]-[0,117])	0,179
Female	-0,330	0,033	([-0,393]-[-0,266])	<0,001
Waist circumference (*R*^2^ = 0,359; *p* < 0,001)				
Age	0,110	0,030	([0,051]-[0,170])	<0,001
Year	0,009	0,030	([-0,051]-[0,068])	0,778
Female	-0,586	0,028	([-0,641]-[-0,531])	<0,001
Hip circumference (*R*^2^ = 0,009; *p* = 0,062)				
Age	0,043	0,038	([-0,031]-[0,117])	0,254
Year	0,017	0,038	([-0,058]-[0,091])	0,660
Female	-0,082	0,035	([-0,150]-[-0,014])	0,019
WHR index (*R*^2^ = 0,351; *p* < 0,001)				
Age	0,054	0,030	([-0,006]-[0,113])	0,079
Year	0,014	0,031	([-0,046]-[0,074])	0,651
Female	-0,587	0,028	([-0,643]-[-0,532])	<0,001

*β*: coefficient of regression between variables; SD: standard deviation; CI: confidence interval; *p*: value used to determine statistical significance; *R*^2^: coefficient of determination.

**Table 5:** Statistical characteristics (mean ± SD) of fitness tests outcomes in research participants.

Years of study	Cooper test (m)		2 km march		Turn right		Turn left	
	Women	Men	Women	Men	Women	Men	Women	Men
2001	2046, 0 ± 197,1	2645, 5 ± 385,3	1636, 5 ± 130,9	1529, 0 ± 172,8	377,1 ± 48, 1	404,7 ± 64, 4	375,0 ± 56, 1	377,3 ± 73, 4
2002	2227, 1 ± 257,2	2552, 5 ± 276,4	1555, 3 ± 122,6	1472, 5 ± 120,8	359,8 ± 43, 8	338,0 ± 38, 5	363,6 ± 57, 9	339,8 ± 44, 0
2003	1882, 8 ± 272,7	2339, 2 ± 342,6	1651, 2 ± 124,0	1498, 8 ± 92, 4	364,6 ± 50, 5	363,2 ± 30, 5	368,5 ± 32, 9	344,0 ± 46, 4
2004	1997, 3 ± 345,5	2322, 1 ± 373,9	1596, 3 ± 133,7	1547, 4 ± 154,4	357,3 ± 40, 2	305,3 ± 61, 6	348,9 ± 36, 3	356,3 ± 63, 3
2005	1937, 4 ± 287,7	2370, 9 ± 508,3	1602, 7 ± 109,5	1391, 6 ± 306,6	348,1 ± 28, 3	364,1 ± 48, 1	356,0 ± 35, 1	369,8 ± 31, 8
2006	1818, 5 ± 233,4	2268, 7 ± 279,1	1599, 7 ± 113,5	1462, 3 ± 88, 6	348,4 ± 30, 8	335,0 ± 57, 8	350,8 ± 32, 9	330,6 ± 60, 4
2007	1907, 9 ± 356,3	2214, 3 ± 365,5	1587, 6 ± 123,2	1477, 5 ± 78, 4	352,6 ± 48, 2	356,0 ± 43, 0	345,6 ± 43, 1	355,3 ± 55, 3
2008	2119, 9 ± 276,9	2574, 9 ± 382,0	1546, 8 ± 57, 3	1461, 2 ± 94, 5	343,1 ± 32, 3	360,2 ± 44, 7	348,7 ± 33, 9	373,9 ± 55, 9
2009	2145, 8 ± 255,1	2660, 9 ± 333,0	1637, 8 ± 97, 3	1562, 4 ± 103,9	347,4 ± 49, 5	379,0 ± 63, 1	353,9 ± 46, 4	377,6 ± 46, 7
2010	2193, 3 ± 178,3	2686, 5 ± 261,7	1589, 3 ± 90, 1	1463, 8 ± 118,0	344,1 ± 53, 6	375,8 ± 61, 3	345,8 ± 48, 2	375,9 ± 53, 6
All	2046, 9 ± 295,8	2519, 1 ± 380,0	1602, 4 ± 110,8	1502, 4 ± 134,2	351,8 ± 44, 2	365,2 ± 56, 4	353,5 ± 42, 8	367,4 ± 52, 5

Mean: average age of student groups surveyed; SD: standard deviation.

**Table 6:** Characteristics of covariance models assessing the impact of research year, gender and age of participants on their fitness tests outcomes.

Feature	*β*	SD	95% CI	*p*
Cooper test (*R*^2^ = 0,346; *p* < 0,001)				
Age	-0,134	0,031	([-0,194]-[-0,075])	<0,001
Year	0,099	0,031	([0,039]-[0,159])	0,001
Female	-0,548	0,028	([-0,604]-[-0,493])	<0,001
2 km march (*R*^2^ = 0,130; *p* < 0,001)				
Age	-0,010	0,035	([-0,079]-[0,059])	0,775
Year	-0,003	0,035	([-0,073]-[0,067])	0,932
Female	0,360	0,033	([0,296]-[0,424])	<0,001
Spinning coordination–turn right (*R*^2^ = 0,024; *p* < 0,001)				
Age	-0,060	0,037	([-0,133]-[0,013])	0,109
Year	-0,098	0,038	([-0,172]-[-0,025])	0,009
Female	-0,139	0,035	([-0,207]-[-0,071])	<0,001
Spinning coordination–turn left (*R*^2^ = 0,023; *p* < 0,001)				
Age	-0,055	0,037	([-0,128]-[0,018])	0,141
Year	-0,059	0,038	([-0,133]-[0,014])	0,114
Female	-0,144	0,035	([-0,212]-[-0,076])	<0,001

*β*: coefficient of regression between variables; SD: standard deviation; CI: confidence interval; *p*: value used to determine statistical significance; *R*^2^: coefficient of determination.

**Table 7:** Calculated Mollison indicators assessing dimorphic differences in motor and anthropometric characteristics.

Years of study	Body height	Body mass	Cooper test	2 km march	Turn right	Turn left
2001	-2,88	-1,72	-1,55	0,62	-0,47	-0,04
2002	-2,69	-2,75	-1,18	0,68	0,57	0,54
2003	-3,10	-2,58	-1,33	1,65	0,05	0,53
2004	-3,60	-2,37	-0,87	0,32	0,84	-0,12
2005	-1,73	-0,89	-0,85	0,69	-0,33	-0,43
2006	-1,01	-1,27	-1,61	1,55	0,23	0,33
2007	-2,07	-1,38	-0,84	1,40	-0,08	-0,17
2008	-1,90	-2,08	-1,19	0,91	-0,38	-0,55
2009	-2,35	-1,58	-1,55	0,72	-0,50	-0,51
2010	-1,78	-1,87	-1,88	1,06	-0,52	-0,56
All	-2,04	-1,68	-1,24	0,74	-0,24	-0,26

Mollison index (magnitude of dimorphic differences of the examined features). WD = (M♀ − M♂)/S♂; M♀: arithmetic mean of the characteristics of the examined group of women; M♂: arithmetic mean of the characteristics of the studied group of men; S♂: standard deviation of the characteristics of the examined group of men.

## Data Availability

Data on individual measurements of anthropometric and motor features used to support the findings of this study may be made available on the researcher's request submitted to the Bioethics Committee of the Nicolaus Copernicus University at the Collegium Medicum Ludwik Rydygiera in Bydgoszcz, which you can contact, ul. M. Curie Skłodowskiej 9, 85-094 Bydgoszcz, Poland, tel. 48 52 585-35-63, fax 52 585-38-11, e-mail: kom.bioetyczna @ http://cm.umk.pl
